# Transcatheter aortic valve implantation for structural valve deterioration of homograft surgical aortic valve using SAPIEN3 Ultra RESILIA: a case report

**DOI:** 10.1093/ehjcr/ytae126

**Published:** 2024-03-11

**Authors:** Kazuki Mizutani, Masafumi Ueno, Genichi Sakaguchi, Gaku Nakazawa

**Affiliations:** Division of Cardiology, Department of Medicine, Kindai University Faculty of Medicine, Ohno-Higashi, Osakasayama, Osaka 589-8511, Japan; Division of Cardiology, Department of Medicine, Kindai University Faculty of Medicine, Ohno-Higashi, Osakasayama, Osaka 589-8511, Japan; Department of Cardiovascular Surgery, Kindai University Faculty of Medicine, Osaka, Japan; Division of Cardiology, Department of Medicine, Kindai University Faculty of Medicine, Ohno-Higashi, Osakasayama, Osaka 589-8511, Japan

**Keywords:** Aortic stenosis, Aortic regurgitation, Case report, Homograft, Structural valve deterioration, Transcatheter aortic valve replacement

## Abstract

**Background:**

There are a few case reports regarding transcatheter aortic valve implantation (TAVI) for deteriorated surgical homograft.

**Case summary:**

We present a case of severe structural valve deterioration (SVD) of homograft surgical aortic valve presenting severe aortic regurgitation in an 84-year-old man with decompensated heart failure. We performed TAVI in homograft valve using 23 mm SAPIEN3 Ultra RESILIA. The resulting grade of paravalvular regurgitation was trace, the post-operative effective orifice area (EOA) was 1.66 cm^2^ (index EOA: 1.19 cm^2^/m^2^), and device success was achieved.

**Discussion:**

Stented bioprosthetic valves are more commonly implanted than mechanical and stentless bioprosthetic valves. In the 1980s and the early 1990s, homografts became particularly popular as alternatives to stented valves. There are several reports of TAVI for homograft SVD, but the paravalvular leakage grade is worse than that of redo-surgical aortic valve replacement, although the mortality rate is lower. However, the valves used in these reports were from older valves such as SAPIEN XT or SAPIEN3. There are no reports using SAPIEN3 Ultra RESILIA with a significant reduction in paravalvular leak due to an external textured polyethylene terephthalate skirt extending 40% higher above the valve inflow than the classical SAPIEN3, which is now available. Transcatheter aortic valve implantation using SAPIEN3 Ultra RESILIA showed good therapeutic efficacy.

**Learning points**

- Fewer patients are undergoing surgical aortic valve replacement with homograft, and we are seeing fewer patients with homograft complicated by severe structural valve deterioration.
- Transcatheter aortic valve in surgical aortic valve for homograft is a feasible and good treatment option.

## Introduction

Transcatheter aortic valve implantation (TAVI) for failing surgical aortic bioprosthetic valves has been recognized as a valid therapeutic option for patients with severe structural valve deterioration (SVD).^1–4^ Currently, stented bioprosthetic valves are more commonly implanted than mechanical and stentless bioprosthetic valves.^5^ In the 1980s and the early 1990s, homografts became particularly popular as alternatives to stented valves.^6^ However, long-term freedom from SVD of homograft is poor, and many cases require re-intervention.^7^

## Summary figure

Aortic valve homograft image used with permission from Netter Images (https://www.netterimages.com).

**Figure:**
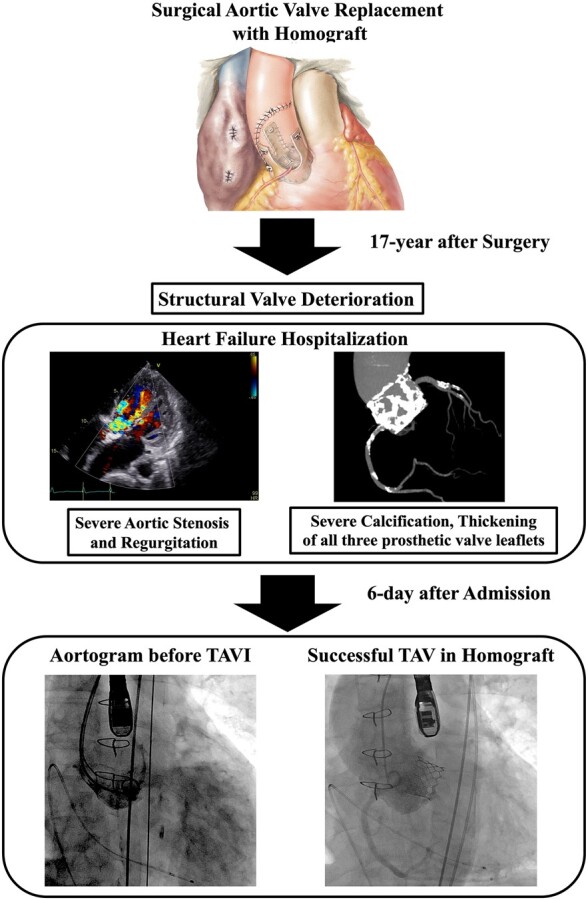


## Case presentation

**Figure 1:**
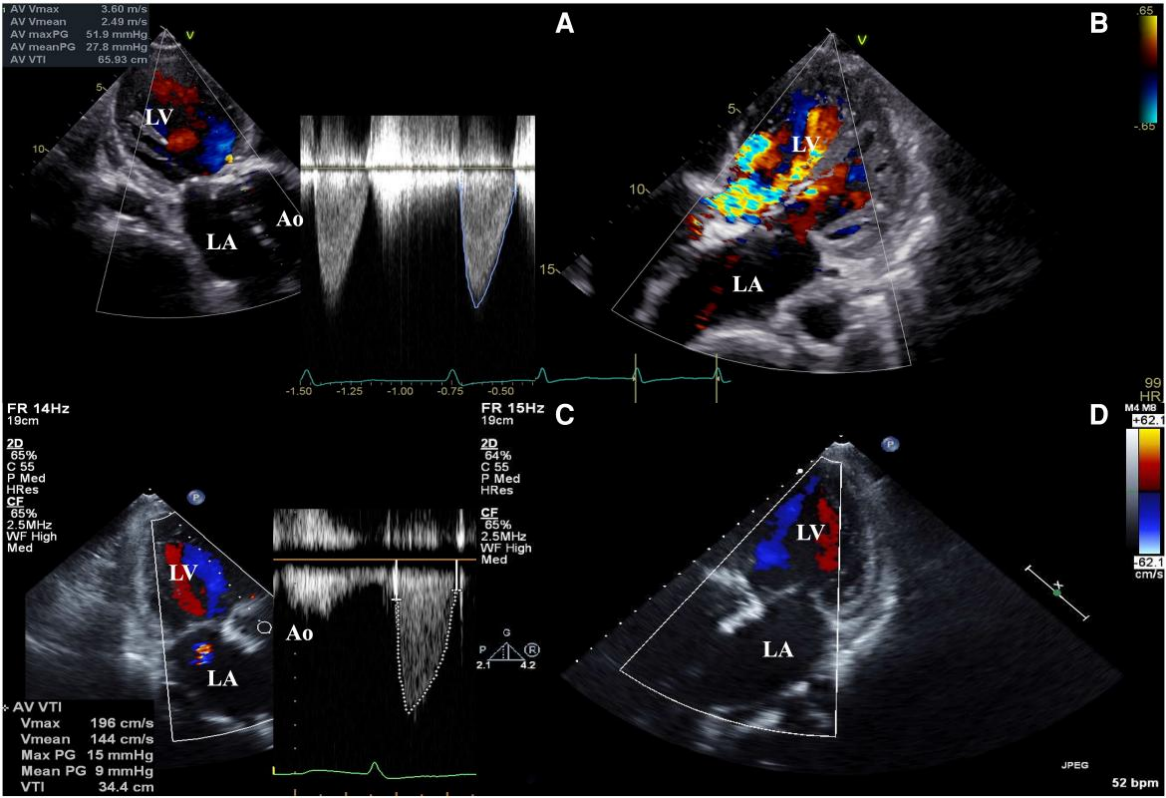
Periprocedural echocardiography. (*A*) Aortic valve assessment using continuous wave Doppler before TAVI. (*B*) AR grade before TAVI. (*C*) Aortic valve assessment using continuous wave Doppler after TAVI. (*D*) AR grade after TAVI. AR, aortic regurgitation; LA, left atrium; LV, left ventricle; TAVI, transcatheter aortic valve implantation.

**Figure 2:**
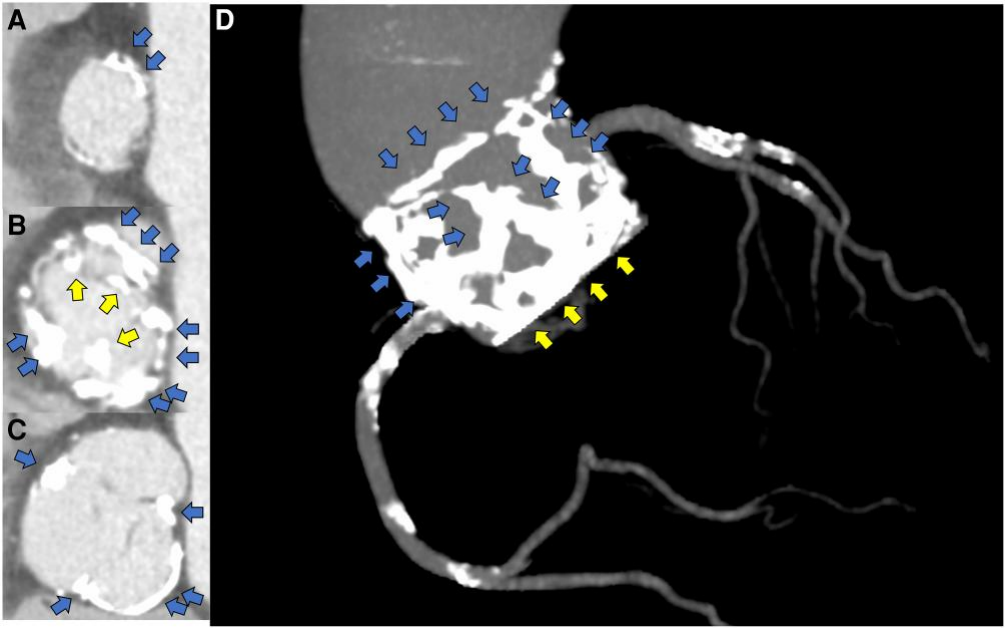
Pre-procedural computed tomography. (*A*) Annulus level, (*B*) 4 mm above the annulus, (*C*) sinus of Valsalva, and (*D*) 3D constructed aortic image. Blue arrows show the calcification of anastomosis. Yellow arrows show leaflet calcification. D, dimension.

**Figure 3:**
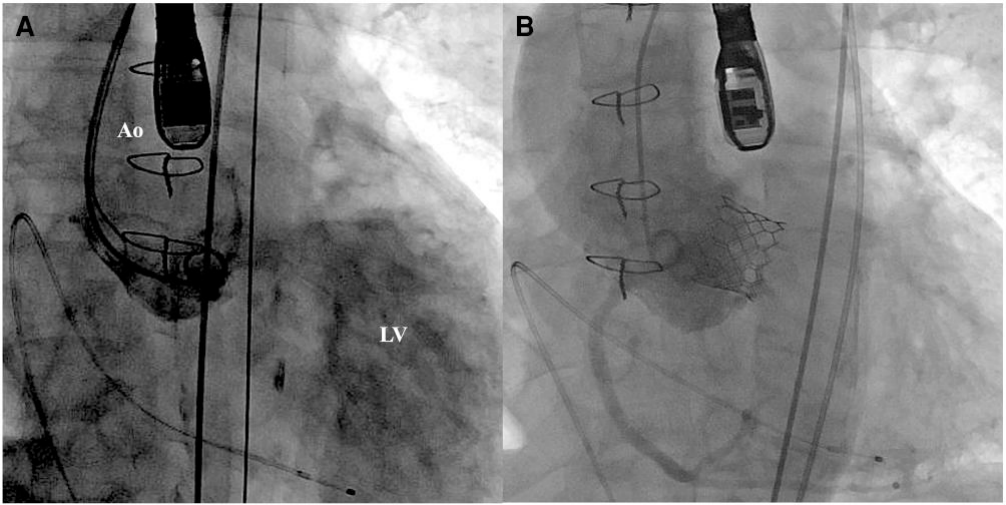
TAVI with 23 mm SAPIEN3 Ultra RESILIA. (*A*) Aortogram before TAVI. (*B*) Aortogram after TAVI. Ao, aorta; LV, left ventricle; TAVI, transcatheter aortic valve implantation.

An 84-year-old man with comorbidities of chronic atrial fibrillation and stage 3b chronic kidney disease was transferred to our hospital with refractory heart failure due to severe SVD and severe aortic stenosis and regurgitation (ASR) of the homograft aortic valve. In 1996, he underwent surgical aortic valve replacement (SAVR) using a 25 mm homograft for severe aortic regurgitation (AR) in the bicuspid valve. The homograft was implanted using the subcoronary method. In 2015, echocardiography detected severe transvalvular regurgitation and elevated estimated right ventricular pressure with moderate tricuspid regurgitation. Since no subjective symptoms such as shortness of breath were observed, outpatient follow-up was performed. However, in 2020, the patient was hospitalized because of worsening heart failure. Heart failure was initially relieved with diuretics, but shortness of breath persisted, requiring three hospitalizations for worsening heart failure by 2023. Furthermore, after oral treatment with azosemide 60 mg and tolvaptan 15 mg, low doses of dobutamine did not alleviate the heart failure (New York Heart Association functional classification: III), and orthopnoea remained at the time of transfer. Other medications he was taking on admission included carvedilol 1.25 mg, spironolactone 25 mg, and edoxaban 30 mg. His blood pressure was 108/48 mmHg, pulse rate was 109 b.p.m., and SPO2 was 94% for indoor air. Physical examination revealed a Levine 3/6 to and fro murmur with the strongest point at the left margin of the left second intercostal sternum. Serum creatinine level was 1.38 mg/dL (normal range 0.65–1.09 mg/dL), haemoglobin level was 8.7 g/dL (normal range 11.0–18.0 mg/dL), and brain natriuretic peptide was 582 pg/mL (normal range ≦ 18.4 pg/mL). Echocardiography revealed severe transvalvular regurgitation with pan-diastolic blood flow in the abdominal aorta suggestive severe AR. Furthermore, it revealed stiffening, shortening, and complete reduction mobility of the non-coronary cusp side leaflet of the homograft valve, which led to poor coaptation of valve leaflets, resulting in severe ASR with an mAVPG of 32 mmHg and an effective orifice area (EOA) of 1.08 cm^2^ (index EOA 0.74 cm^2^/m^2^) (*Figure 1A* and *B*). Our heart team decided to perform transcatheter aortic valve (TAV) in surgical aortic valve (SAV) because his clinical frailty was 5 and the Society of Thoracic Surgeons predicted mortality risk score was 15.8%. Contrast-enhanced computed tomography (CT) revealed severe calcification, thickening of all three prosthetic valve leaflets, calcified fusion in right- and non-coronary cusp (*Figure 2B*), and calcification of the anastomosis of the homograft with the autologous artery (*Figure 2A–D*). The area and perimeter of annulus plane was 420.3 mm^2^ and 76.6 mm, respectively. The sinus of Valsalva (37.5 × 39.4 × 42.7 mm) and sino-tubular junction (46.5 × 37.1 mm) were large enough that there was no concern about coronary artery occlusion risk and Valsalva injury due to TAV in SAV, and the minimum vessel diameter for lower extremity arterial access was 6.5 mm. The CT findings suggested that TAVI with a balloon-expandable valve via transfemoral access could be safely performed. In addition, in this case, the self-expandable TAVR was expected to be difficult to implant accurately due to the lack of calcification of the prosthetic valve leaflets. Although we could not find any reports on the exact valve sizing in TAVI for homograft, we chose 23 mm in this case in accordance with the sizing of TAVI for autologous valves. Following a rapid multidisciplinary team discussion, we performed urgent TAVI using a 23 mm SAPIEN3 Ultra RESILIA (Edwards Lifesciences, Irvine, CA, USA) (*Figure 3A* and *B* and Supplementary material online, *Videos S1* and *S2*). The resulting grade of paravalvular regurgitation was trace, the post-operative EOA was 1.66 cm^2^ (index EOA: 1.19 cm^2^/m^2^), and device success was achieved (*Figure 1C* and *D*). The patient’s post-operative course was good. Post-operative echocardiography showed good prosthetic valve function with mAVPG of 12 mmHg, an EOA of 1.68 cm^2^ (index EOA 1.15 cm^2^/m^2^), and with trace paravalvular leakage. Serum brain natriuretic peptide level decreased to 114 pg/mL. He was able to discontinue dobutamine and was discharged from the hospital on the 14th post-operative day without any other major complications.

## Discussion

Stented bioprosthetic valves are more commonly implanted than mechanical and stentless bioprosthetic valves.^5^ In the 1980s and the early 1990s, homografts became particularly popular as alternatives to stented valves. However, homograft use has declined due to the complexity of implantation, concerns about premature valve structural deterioration, and the high risk of reoperation.^6,8^ Freedom from SVD, defined as an AR grade of mild or greater or a mean transvalvular pressure gradient of 20 mmHg or greater at 8 years, was reported in 37% of the homograft group.^6^ In the present case, homograft SVD was thought to have occurred 9 years post-operatively. The results of redo-SAVR for homograft SVD were poor, with a reported 30-day mortality rate of 8%.^9^

Conversely, there are several reports of TAVI for homograft SVD, but the paravalvular leak (PVL) grade is worse than that of redo-SAVR, although the mortality rate is lower.^9,10^ Generally, in SAVR using homograft, unlike the use of other stented or stentless bioprosthetic valves, anastomosis is performed at the level of the annulus basal plane of the patient’s own annulus, so when SVD occurs, calcification is also observed at the anastomotic site, as in this case, and this may be one of the reasons for the increase in PVL when TAV in SAV is performed. However, the valves used in these reports were from older valves such as SAPIEN XT (Edwards Lifesciences, Irvine, CA, USA) or SAPIEN3 (Edwards Lifesciences, Irvine, CA, USA). There are no reports using SAPIEN3 Ultra RESILIA (Edwards Lifesciences, Irvine, CA, USA) with a significant reduction in PVL due to an external textured polyethylene terephthalate skirt extending 40% higher above the valve inflow than the classical SAPIEN3, which is now available. In particular, the textured and improved skirt may contribute to better PVL suppression, as well as better crimping of calcification at the annulus anastomosis of the homograft. Therefore, TAV in homograft SAV using SAPIEN3 Ultra RESILIA showed good therapeutic efficacy, and regardless of the context in which homograft were used in SAVR, TAV in SAV is likely to be a valid treatment option.

## Lead author biography


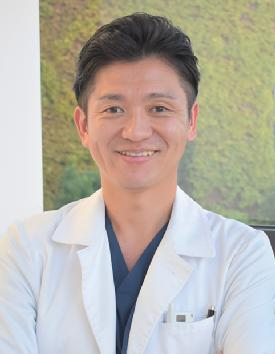


Kazuki Mizutani specializes in structural heart intervention and has performed 800 TAVI procedures.

## Supplementary Material

ytae126_Supplementary_Data

## Notes

**Consent:** In preparing this case report, the authors confirm that written consent for submission and publication on social media of this case report, including images and associated texts, has been obtained from the patient in line with the COPE guidelines.

**Funding:** None declared.

**Data availability:** The data underlying this article are available in the article and in its online Supplementary material.
